# Biological and biochemical properties of two *Xenopus laevis N*-acetylgalactosaminyltransferases with contrasting roles in embryogenesis

**DOI:** 10.1016/j.cbpb.2014.10.003

**Published:** 2015-02

**Authors:** Josef Voglmeir, Nicolas Laurent, Sabine L. Flitsch, Michael Oelgeschläger, Iain B.H. Wilson

**Affiliations:** a Department für Chemie, Universität für Bodenkultur, Wien, Austria; b Manchester Interdisciplinary Biocentre, University of Manchester, UK; c Max-Planck-Institut für Immunbiologie, Freiburg, Germany

**Keywords:** xGalNAc-T, *Xenopus N*-acetylgalactosaminyltransferase, *N*-acetylgalactosaminyltransferase, Mucin, Development

## Abstract

The biosynthesis of mucin-type O-linked glycans in animals is initiated by members of the large family of polypeptide *N*-acetylgalactosaminyltransferases (GalNAc-Ts), which play important roles in embryogenesis, organogenesis, adult tissue homeostasis and carcinogenesis. Until now, the mammalian forms of these enzymes have been the best characterized. However, two N-acetylgalactosaminyltransferases (xGalNAc-T6 and xGalNAc-T16) from the African clawed frog (*Xenopus laevis*), which are most homologous to those encoded by the human GALNT6 and GALNT16 (GALNTL1) genes, were shown to have contrasting roles in TGF-β/BMP signaling in embryogenesis. In this study we have examined these two enzymes further and show differences in their in vivo function during *X. laevis* embyrogenesis as evidenced by in situ hybridization and overexpression experiments. In terms of enzymatic activity, both enzymes were found to be active towards the EA2 peptide, but display differential activity towards a peptide based on the sequence of ActR-IIB, a receptor relevant to TGF-β/BMP signaling. In summary, these data demonstrate that these two enzymes from different branches of the *N*-acetylgalactosaminyltransferase do not only display differential substrate specificities, but also specific and distinct expression pattern and biological activities in vivo.

## 1 Introduction

There are various molecular markers distinguishing animals from plants, fungi, protists and bacteria. Amongst these are glycan modifications of peptides and proteins which appear to be rather restricted to the Metazoan lineage—such as proteoglycans based on *O-*linked xylose (Xyl) linked to serine or mucins based on *O-*linked *N-*acetylgalactosamine (GalNAc) linked to serine or threonine. These types of animal-specific glycosylation are also strongly evolutionarily conserved throughout the animal kingdom, indicating a specific and fundamental role in animal development and physiology. Indeed, gel-forming mucins emerged early during metazoal evolution and are found, e.g., in the cnidaria (Lang et al., 2007). The modification of proteins by GalNAc residues is initiated by the action of a family of *N*-acetylgalactosaminyltransferases (ppGalNAc-T, ppGaNTase or GalNAc-T enzymes encoded by *GALNT* genes in vertebrates; EC 2.4.1.41). Recent data from model organisms such as *Drosophila melanogaster* and *Xenopus laevis*, as well as the discovery of the basis of familial tumoral calcinosis, have indicated that the modification of proteins by *N-*acetylgalactosamine to form ‘mucin-type’ O-linked oligosaccharides is required for normal animal development. For instance, lethal mutations are associated with two *D. melanogaster N*-acetylgalactosaminyltransferase genes (*pGaNT35A* and *pGaNT5*) (Ten Hagen and Tran, 2002; Tran et al., 2012), whereas the association of mutations in the human *GALNT3* gene with familial tumoral calcinosis also highlights the importance of this type of protein modification (Topaz et al., 2004). Furthermore, it has been suggested that the modification of fibronectin by mammalian GalNAc-T6 may have a role in breast cancer development and progression (Park et al., 2011).

The in silico prediction of which sites may become O-glycosylated proved difficult and only rather vague ‘consensus’ sequences for this modification were proposed (Wilson et al., 1991; O'Connell et al., 1992; Hansen et al., 1995); many assays with partially-purified enzymes were also performed in order to define these sequences by in vitro means (Cottrell et al., 1992; O'Connell et al., 1992). In retrospect, it is obvious that such attempts at defining consensus sequences would fail, due to the subsequent isolation of a large number of cDNAs encoding *N*-acetylgalactosaminyltransferase isoforms from mammalian, insect and nematode sources; in particular, many GalNAc-T isoforms have been identified by, especially, the Tabak, Clausen and Narimatsu laboratories (Bennett et al., 2012). Over the years, overlapping substrate specificities, requirements for pre-glycosylated substrates and variable gene expression patterns have been determined for the twenty or so mammalian members of this enzyme family. Activity has been reported for some seventeen mammalian *N*-acetylgalactosaminyltransferases, the latest being for GalNAc-Ts 15–18 (Cheng et al., 2004; Peng et al., 2010; Raman et al., 2012). Data are still awaited to define the specificities of human GalNAc-T8, -T19, and -T20; potentially GalNAc-T18, a member of the vertebrate-specific Y subfamily, modulates the activity of other members of this enzyme family (Li et al., 2012), although a low activity was detected in a different study (Raman et al., 2012). Considering the partial functional redundancy of these enzymes, a challenge has been to match the biochemical knowledge with their biological significance; this has also been complicated by differences in nomenclature and numbering between different laboratories.

In a previous study it was shown that an *N*-acetylgalactosaminyltransferase from *X. laevis* plays an important role in the regulation of early embryogenesis (Herr et al., 2008). The gene was described to display the highest identity with the human *GALNTL1* (*N-*acetylgalactosaminyltransferase-like 1 gene, now known as *GALNT16*; the enzyme is hereafter defined as xGalNAc-T16). Specifically, this amphibian GalNAc-T16 was shown to regulate the TGF-β/BMP (tissue growth factor-β/bone morphogenetic protein) pathway during early embryonic development, potentially modifying and modulating the activity of a TGF-β receptor known as ActR-IIB (Activin A receptor type IIB) (Herr et al., 2008). A second amphibian *N*-acetylgalactosaminyltransferase displaying the highest homology to human GalNAc-T6 also shows a relationship to BMP signaling, since its in vivo expression during embryogenesis coincides with high endogenous BMP signaling and is also dependent on the activity of the BMP pathway. xGalNAc-T6 and xGalNAc-T16 are highly homologous to isoforms in other species, e.g. the human isoforms GalNAc-T6 and GalNAc-T16 both show > 70% sequence similarity to the frog isoforms (Herr et al., 2008).

Whereas the mammalian forms of *N*-acetylgalactosaminyltransferases have been generally well characterized in terms of their enzymology, there are few data on amphibian forms of these enzymes; considering the developmental significance of xGalNAc-T6 and xGalNAc-T16, it was sought to biochemically characterize these upon expression in *Pichia pastoris*. Our biochemical analysis of these two proteins would suggest that ActR-IIB is indeed an excellent substrate for xGalNAc-T16, but not xGalNAc-T6. In addition to the distinct and specific substrate specificities of these two *N*-acetylgalactosaminyltransferases, they also display complex but very different expression pattern in the early Xenopus embryo as well as different biological activities in overexpression studies. This comprehensive study, thereby, demonstrates differential biochemical and biological functions of two *N-*acetylgalactosaminyltransferases during early vertebrate embryogenesis.

## 2 Experimental procedures

### 2.1 Developmental studies

In vitro fertilization, embryo and explant culture, microinjection of synthetic mRNA, in situ hybridization and RT-PCR analysis were performed as described (Herr et al., 2008). Full-length cDNA IMAGE consortium clones encoding xGalNAc-T6 and xGalNAc-T16 were obtained from the RZPD (see above) and the open reading frames cloned by PCR into pCS2 +. For mRNA synthesis, the constructs were cut with NotI and transcribed with SP6 RNA polymerase using the mMessage mMachine Kit (Ambion, Austin, TX). Probes for in situ hybridization were directly generated from the GalNAc-T6 and -T16 cDNA clones cut with KpnI and BamHI, respectively and transcribed with T7 polymerase. For RT-PCR analysis, embryos were injected in the animal pole of all four blastomeres at the 4-cell stages with 100 pg noggin or 400 pg op1 mRNA, explants isolated at stage 9 and cultured until stage 24. The primers for the RT-PCR analysis of ectodermal explants were described elsewhere (Herr et al., 2008). Expression of α*-actin* served as a control for mesoderm contamination and *odc* as a loading control.

### 2.2 *Cloning of* X. laevis *N-acetylgalactosaminyltransferase cDNA fragments*

Using two previously-described full length clones as templates (Herr et al., 2008), fragments of the cDNAs encoding the predicted lumenal regions of the *X. laevis* xGalNAc-T6 and xGalNAc-T16 (respectively previously designated as xGalnt-6 and xGalntl-1) were isolated by PCR using KOD Polymerase and two primer pairs: xGalnt-6/1/EcoRI (cggaattcaaagatgttgggaatggag) with xGalnt-6/2/XbaI (tgctctagatttagctgaaaccccagc) and xGalnt-16/1/EcoRI (cggaattccaggacagcaagcccca) with xGalnt-16/2/XbaI (tgctctagactcatgtagcctgaagtatc). The templates were two IMAGE EST clones (BC110706 and BM192636) obtained from RZPD (now SourceBioscience, Berlin); the isolated PCR fragments were purified using a GFX kit (GE Healthcare) and digested with EcoRI and XbaI prior to ligation into pPICZαFLAGC3 (a variant of the Invitrogen pPICZαC plasmid prepared by inverse PCR to generate a form encoding an N-terminal FLAG tag) cut with the same enzymes (Nemčovičová et al., 2013). Positive clones were verified by DNA sequencing and linearized before transformation into *P. pastoris* GS115. Phylogenetic studies were based on comparing GalNAc-T isoforms of frog, human, bovine, mouse, chicken and zebrafish origin using one-click analysis on phylogeny.fr (Dereeper et al., 2008).

### 2.3 Recombinant expression of N-acetylgalactosaminyltransferases

Pre-cultures of selected independent recombinant *P. pastoris* clones were set up in BMGY medium containing 100 μg/mL zeocin and inoculated overnight at 30 °C with continuous shaking (250 rpm). The cells were then collected by centrifugation (10 min, 1500 *g*, 4 °C), washed and resuspended in the induction medium containing 1% methanol (BMMY medium) to an OD_600_ value of 1.0; depending on the scale of fermentation, 50 or 400 mL of these suspensions was transferred into fresh fermentation flasks, and incubated further at 30 °C with continuous shaking (250 rpm). After 24, 48 and 72 h additional methanol was added to maintain the concentration of 1% (*v/v*), and the induction temperature was decreased to 16 °C. 24 h after the last induction with methanol, the cultures were centrifuged at 4000 *g* for 10 min at 4 °C; for large-scale fermentations, residual particles in the culture supernatant were removed using a filter syringe (0.45 μm pore size). The supernatants were then concentrated approximately 100-fold (using centrifugal concentrators with M_R_ = 30 kDa cut-off), and stored in 60 μL-aliquots in the − 80 °C freezer. Expression of the *N*-acetylgalactosaminyltransferases was proven by tryptic peptide mass fingerprinting as well as by Western blotting with monoclonal anti-FLAG antibody (Sigma Aldrich).

### 2.4 Protein quantification using bicinchoninic acid (BCA)

Protein quantification was performed using a microplate BCA protein assay (Thermo Pierce). Concentrated supernatant containing active xGalNAcT isoforms was quantified with the BCA assay in undiluted, 1:5 and 1:25 dilutions, and the absorbance was analysed at 562 nm, and compared with the absorbance of BSA-standard concentrations (25–2000 μg/mL BSA). The protein concentration was 17.9 mg/mL for xGalNAc-T6 and 6.7 mg/mL for xGalNAc-T16, respectively.

### 2.5 Assays for N-acetylgalactosaminyltransferase activity

Typically, enzyme activity was tested using a mixture of 1 mM peptide (for sequences see Table 1), 1.5 mM UDP-GalNAc, 20 mM MES (pH 7.5), 10 mM MnCl_2_, 1 U CIP (Calf Intestinal Alkaline Phosphatase, NEB) and 0.4 μg BSA (Bovine Serum Albumin, NEB) with 2 μL enzyme solution in a total of 10 μL incubated at 37 °C for the times indicated in the figure legends; CIP was added in order to avoid inhibition by the buildup of UDP (Fritz et al., 2004), thereby mimicking Golgi apyrase (Gao et al., 1999), while BSA was observed in preliminary experiments to stabilise the enzymes. The amount of enzyme added was adjusted to ensure a linear relationship of product formation over time. Incorporation of GalNAc into peptides was determined by both MALDI-TOF MS and by HPLC. For the latter, an Agilent 1200 LC-system equipped with a reversed phase ODS Hypersil 5 μm column (4 × 250 mm) was used. Solvent A was 0.1% trifluoroacetic acid (TFA) in water, and solvent B was 0.1% TFA in acetonitrile and mixed at a flow rate of 1 mL/min according to the following gradient program: 5% B for the first 5 min, then a linear gradient from 5–25 min of 5–15%, followed by a wash step and then a return to the initial conditions. Elution of the unglycosylated and glycosylated forms of the peptides was monitored at 210 nm.

### 2.6 MALDI-ToF MS/MS analysis

Assays were performed using the same basic mixture as described above, except that 1 mM of an adapted form of the EA2 peptide with an C-terminal arginine rather than lysine residue (PTTDSTTPAPTTR) was employed. The products were analysed using a Bruker Autoflex Speed MALDI ToF/ToF mass spectrometer (equipped with a Smartbeam™-II laser) in positive mode; the samples were first dried under vacuum on a steel plate and then 6-aza-2-thiothymine (ATT) was applied twice as matrix. Using the LIFT mode, fragmentation of parent ions was performed by laser-induced dissociation and 4000 shots were sampled. MS/MS spectra were processed with the manufacturer's software (Bruker Flexanalysis 3.3.80) using the SNAP algorithm with a signal/noise threshold of 3 (four-times smoothed).

## 3 Results

### 3.1 Developmental studies on two Xenopus GalNAc-T genes

**Fig. 1:**
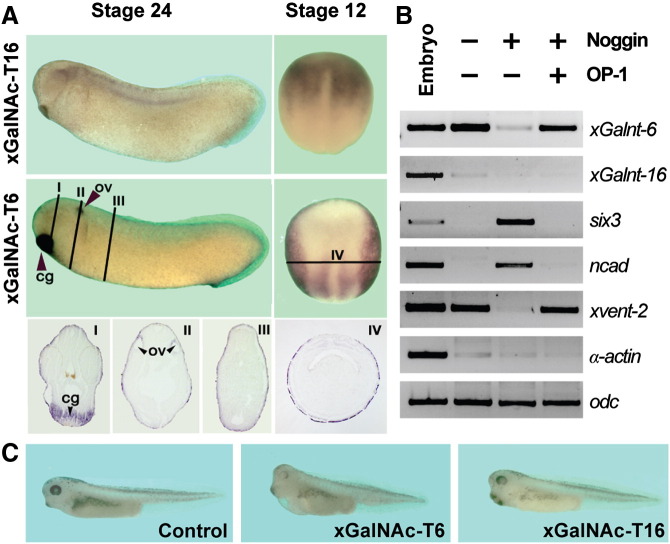
Biological characterization of *Xenopus* xGalNAc-T6 and –T16. (A) Expression analysis by in situ hybridization of stage 12 (dorsal view) and stage 24 *Xenopus* embryos. Whereas xGalNAc-T16 is expressed in the anterior mesoderm and in the deep layer of the lateral neural plate at stage 12 and in anterior brain, neural crest mediolateral spinal cord and notochord at stage 24 (see also Herr et al., 2008), GalNAc-T16 is highly expressed in the ectoderm, but excluded from the neuroectoderm at stage 12 and highly expressed in the otic vesicle (ov) and cement gland (cg) at stage 24. Paraffin sections, at the indicated positions of the stage 24 and stage 12 embryos stained for xGalNAc-T6 mRNA, are shown below. (B) RT-PCR analysis of stage 20 ectodermal explants isolated from embryos microinjected four times animally at the 4–8 cell stage with 20 pg noggin mRNA, 100 pg op-1 mRNA or both. The expression of *six3* and *ncad* indicated neuralization of the explants, *xvent-2* was used as a marker for active BMP signaling, *a-actin* as a marker for mesoderm and *odc* as a loading control. (C) Typical phenotypes of untreated Xenopus embryos and embryos microinjected twice dorsal-animally at the 4–8 cell stage with 400 pg mRNA encoding xGalNAc-T6 or xGalNAc-T16. All microinjection experiments were performed at least three times (*n* > 25) with > 70% of the embryos displaying an reduction of head and eye structures after overexpression of GalNAc-T6 or a expansion of anterior structures, in particular of the cement gland, after GalNAc-T16 injections as described by Herr et al. (2008).

In a previous study, the expression of four different members of the polypeptide N-acetylgalactosaminyltransferase (GalNAc-T) family in *X. laevis* was examined by PCR; one of these genes, xGalNAc-T16 (previously known as xGalntl-1) had been identified during a screen for genes negatively regulated by BMP signals and its tissue-specific expression throughout early *Xenopus* embryogenesis was described (Herr et al., 2008). In *X. laevis*, GalNAc-T16 is expressed dorsally already at gastrula stages, in the anterior mesoderm at neurula stages and in the neural tube, neural crest and notochord at tadpole stages. Biochemical and functional analysis of xGalNAc-T16 suggested that this protein regulates mesoderm formation and neural patterning through regulation of the TGF-β receptor ActR-IIB (Herr et al., 2008). Therefore, we compared the expression of xGalNAc-T16 with xGalNAc-T6 during early *Xenopus* development by in situ hybridization (Fig. 1a). In contrast to xGalNAc-T16, we detected xGalNAc-T6 mRNA specifically in the ventral ectoderm, excluded from the dorsal neuroectoderm, at neurula stages and in the cement gland, otic vesicle and ectoderm at tailbud stages. The expression pattern of xGalNAc-T16 implied transcriptional regulation by active BMP signals. We directly addressed this question by RT-PCR studies in ectodermal explants that, in isolation, differentiate autonomously in epidermal structures (Zimmerman et al., 1996). As expected from the in situ analysis, only the expression of xGalNAc-T6 could be detected in untreated explants. However, this expression was abolished after overexpression of the bona fide BMP antagonist Noggin (Wilson and Hemmati-Brivanlou, 1995) and this effect was rescued after co-injection of BMP7/OP-1 mRNA (Fig. 1b). Thus, the expression of xGalNAc-T6 in ectodermal explants requires active BMP signaling. Finally, animal overexpression of xGalNAc-T16 promotes the development of anterior (head) structures, whereas xGalNAc-T6 overexpression resulted in a slight reduction of these structures (Fig. 1c).

### 3.2 Screening of the peptide specificities by MALDI-ToF MS

**Fig. 2:**
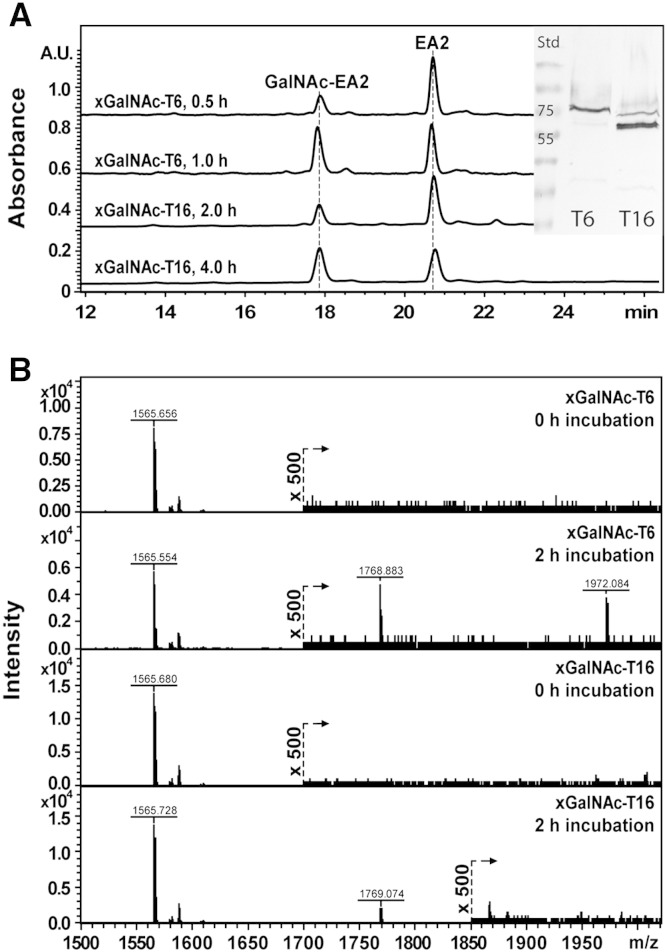
Activity of frog xGalNAc-T6 and –T16 towards the EA2 and ActR-IIB peptides. *A:* The activities of the xGalNAc-T6 (after 30 and 60 min; 3.6 μg protein) and -T16 (after 2 and 4 h; 13.4 μg protein) were tested in vitro towards the EA2 peptide and products were identified by either RP-HPLC as evidenced by the appearance of a peak of lower retention time; the inset is of an anti-FLAG Western blot of the two enzyme preparations. *B:* Incubations of xGalNAc-T6 and -T16 with the ActR-IIB peptide were analysed by MALDI-TOF MS (lower panel; products with *m/z* 1768 and 1972); significant activity (ca. 10% conversion; *m/z* 1769) was observed only with xGalNAc-T16, but traces of singly- and doubly-glycosylated products were observed with xGalNAc-T6 (products of *m/z* 1768 and 1972 were observed with a magnified scaling factor of 500).

The two enzymes, xGalNAc-T6 and xGalNAc-T16, were then examined in terms of their in vitro biochemical function; both enzymes were successfully expressed in the yeast *P. pastoris* as judged by tryptic peptide mapping (data not shown) or Western blotting (Fig. 2) of the recombinant proteins; the latter shows distinct anti-FLAG reactive bands of around 75 kDa for xGalNAc-T6 and 60 kDa for xGalNAc-T16. These apparent molecular weights compare well to the respective theoretical values of 70 kDa (with two potential N-glycosylation sites) and 63 kDa (with no canonical N-glycosylation sites). A panel of peptides was tested in a solution-based assay using MALDI-ToF MS in order to give an overview of the substrate specificity of the two xGalNAcT isoforms (Table 1 and Fig. 2). Peptides derived from mucin-type proteins (Muc1, Muc5, EA2; the latter being a tandem repeat of rat submandibular gland mucin) and artificial mucin-type peptides (OPSS) were used as well as peptides used typically to test the activity of other peptide-*O-*glycosyltransferases, such as RNApII (an *O*-GlcNAc-transferase substrate) or neuroglycan (an *O*-xylosyltransferase substrate). We also tested a peptide based on a portion of the human ActR-IIB receptor (Gly*_123_* to Leu*_137_*); in frog embryo explants, this receptor was shown ex vivo to be directly affected by xGalNAc-T16 (Herr et al., 2008).

In general, xGalNAc-T6 seems to accept a broader range of substrates than xGalNAc-T16, with the exception of the ActR IIB peptide, which is only poorly modified by the former enzyme (an estimated 0.2% over 2 h under the conditions used as compared to 10% by xGalNAc-T16). On the other hand, xGalNAc-T16 incorporates just one GalNAc residue onto seven possible glycosylation sites of the EA2 peptide, whereas xGalNAc-T6 glycosylates up to three sites (Table 1); many other mammalian isoforms incorporate up to five residues per peptide (Pratt et al., 2004). Co-incubation of the EA2 peptide with both enzymes did not result in an increased number of glycosylation events (data not shown). By comparison to other studies, the mammalian GalNAc-T6 has been previously shown to act on the Muc1 and EA2 peptides (Bennett et al., 1999), whereas the very recent data on mammalian GalNAcT-16 has been found to display significant activity towards Muc5 and EA2 (Raman et al., 2012), as well as to two domains of Notch (Boskovski et al., 2013). Preliminary analysis of the kinetics of the two *Xenopus* enzymes towards the peptide acceptors indicated *K_M_* values of 1.0 and 2.6 mM for xGalNAc-T6 and -T16 with the EA2 peptide and of 0.3 mM for xGalNAc-T16 with the ActR-IIB peptide (data not shown); this compares to *K_M_* values for EA2 of between 0.04 and 1 mM of mammalian and invertebrate *N*-acetylgalactosaminyltransferases (Ten Hagen et al., 1998, 1999).

**Fig. 3:**
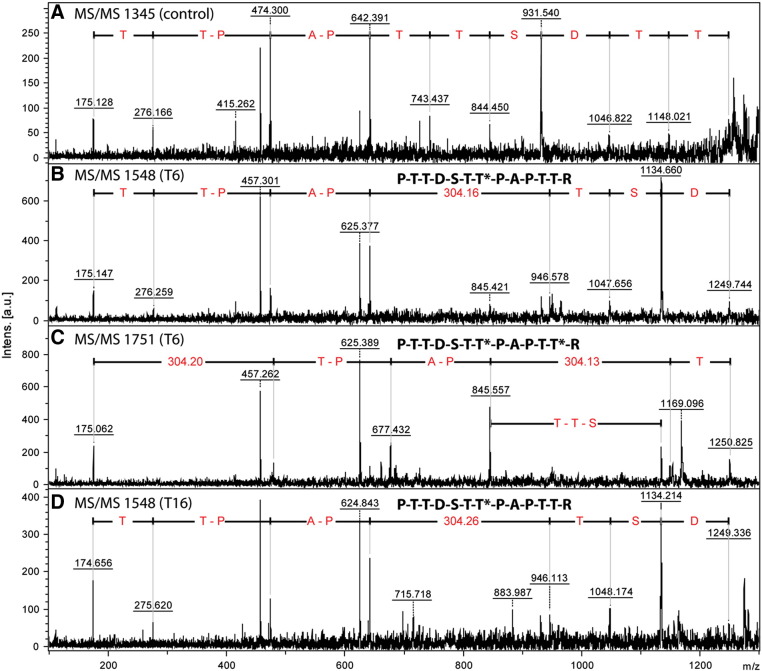
Tandem mass spectrometric analysis of xGalNAcT products. A modified form of the EA2 peptide was employed in a MALDI-ToF-based assay and the fragmentation patterns of the substrate (A; *m/z* 1345), the mono- and di-glycosylated products of xGalNAc-T6 (B and C; *m/z* 1548 and 1751) and the sole product of xGalNAc-T16 (*m/z* 1548) were compared after overnight incubation. Series of putative y-fragments are annotated with amino acids (one-letter code) commencing with the C-terminal arginine residue (y_1_ ion of 175); a mass difference of 304 corresponds to a glycosylated threonine, whereas mass differences of − 17 may result from loss of ammonia from y-ions. The sequences of the glycosylated peptides are shown with the putatively-modified threonines indicated with asterisks.

The products of assays performed with a modified form of the EA2 peptide (PTTDSTTPAPTTR) were analysed by MS/MS in order to define the sites of glycosylation. As judged by the series of probable y-fragments (Fig. 3), xGalNAc-T6 modified first Thr_7_ of this peptide, probably followed by Thr_12_ for the diglycosylated species. On the other hand, xGalNAc-T16 appeared to solely glycosylate Thr_7_ under the assay conditions employed. Previous data on other *N*-acetylgalactosaminyltransferases would suggest that Thr_7_ is indeed a frequently favoured glycosylation site for those enzymes which can accept non-glycosylated peptides as substrates; in the crystal structure of human GalNAc-T2, Thr_7_ of the EA2 peptide is well located to be the glycosylated residue (Fritz et al., 2006). Also, human GalNAc-T1 and T11 as well as *Drosophila* GalNAc-T1 glycosylate either Thr_6_ and/or Thr_7_ (ten Hagen et al., 2001; Schwientek et al., 2002). The preference for xGalNAc-T6 then glycosylating the C-terminal threonine is in line with recent data on its bias towards singly glycosylated peptides where the initial glycosylation has taken place at a more N-terminal site (Gerken et al., 2013).

### 3.3 Temperature optima of xGalNAc-T6 and -T16

**Fig. 4:**
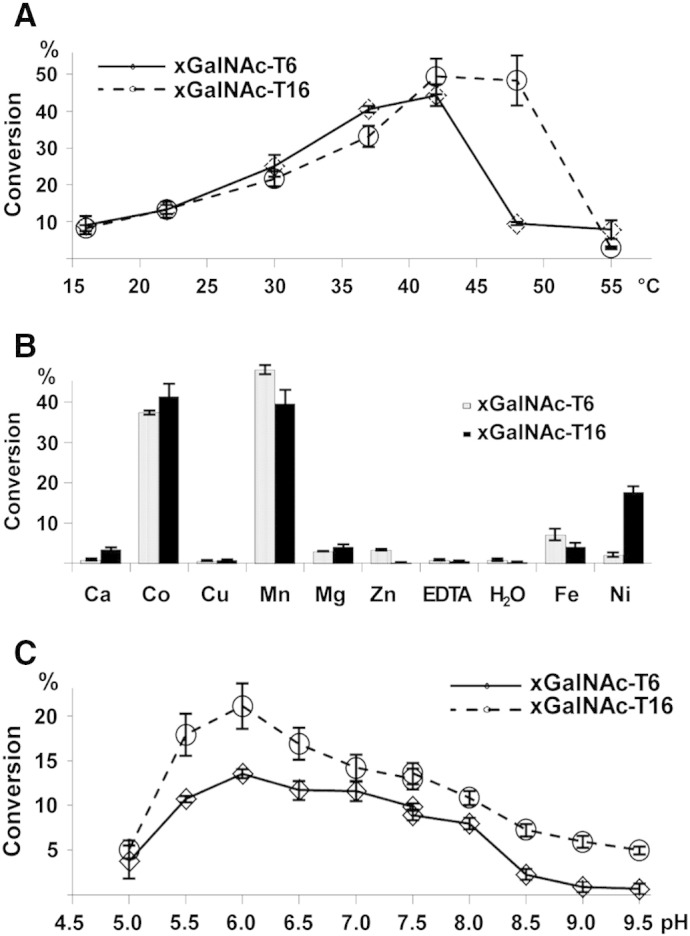
Biochemical characterization of frog xGalNAc-T6 and -T16. The following parameters were measured for both enzymes using the EA2 peptide: (A) temperature optimum, (B) cation dependence and (C) pH optimum. The conditions of time and enzyme dilution were shown for both enzymes to result in the transfer of a single GalNAc residue.

Performing the reaction at different temperatures (16, 22, 30, 37, 42, 48 and 55 °C) with the optimized conditions described above showed that both enzymes had their maximum activity at 42 °C (Fig. 4a). Relatively few data have seemingly been published on the temperature optimum of other recombinant *N*-acetylgalactosaminyltransferases; isoforms from *Caenorhabditis* were most active at 23 °C (Hagen and Nehrke, 1998), similar to data on cestode and fly enzymes (*Echinococcus* GalNAc-T1 at 28 °C and *Drosophila* GalNAc-T3 at 29–33 °C) (Freire et al., 2004; Nakamura et al., 2004). The comparison with the temperature optima of other glycosyltransferases (e.g., the peptide O-xylosyltransferases) suggests that frog enzymes might have a higher temperature optimum than for mammalian or invertebrate enzymes (Brunner et al., 2006; Voglmeir et al., 2007). The reason for this elevated temperature maximum could be that frogs are like all other amphibians *ectotherm*, and have therefore to adjust their body temperature also to many different surroundings (Feder and Burggren, 1992). Although both isoforms reach maximum activity at a temperature of 42 °C, xGalNAc-T6 seems to have a slightly lower optimum than xGalNAcT-16, judged by the lower activity of the enzyme at 48 °C. However, due to reduced stability at higher temperatures as observed in preliminary experiments, the incubation temperature for other experiments was kept at 37 °C.

### 3.4 Metal ion requirements of xGalNAc-T6 and -T16

The metal dependency of many glycosyltransferases of the GT-A superfamily is based on the formation of a complex between a divalent metal ion and the oxygen atoms of the two phosphate groups of the NDP-sugar (Breton et al., 2006). Several studies describe Mn^2 +^ as the best activator for enzymatic activity of *N*-acetylgalactosaminyltransferases, with an optimal concentration between 2.5 and 10 mM (Takeuchi et al., 1985; Wang et al., 1992), whereby reducing the concentration to 0.4 mM results in 50% loss of activity, whereas the increase to 20, 30 and 100 mM decreases the activity to 90%, 50% and 20%, respectively (Wang et al., 1992; Mendicino and Sangadala, 1998). Fig. 4b shows the screening of metal dependencies of the two xGalNAcT-isoforms at a metal ion concentration of 10 mM. Both xGalNAcT isoforms showed comparable conversion rates under the influence of various divalent cations, H_2_O and EDTA. Using H_2_O should reveal the enzyme activity without addition of metal ions, whereas the addition of EDTA is employed to chelate potential residual divalent cations bound to the enzyme or divalent cations present in the enzyme solution (Sugiura et al., 1982). No activities (less than 0.5% conversion rate) were observed with these controls.

In general, the obtained data reflect the results of published studies on mammalian *N*-acetylgalactosaminyltransferases. Mn^2 +^ and Co^2 +^ displayed the strongest stimulation of activity, whereas Ni^2 +^, Fe^2 +^, Mg^2 +^, Ca^2 +^ and Zn^2 +^ also stimulated to a smaller extent. xGalNAc-T6 showed a slightly higher affinity for Mn^2 +^ over Co^2 +^ (78% activity compared to Mn^2 +^), whereas xGalNAcT-16 preferred Co^2 +^ (Mn^2 +^ showed only 96% of the activity compared to Co^2 +^). Studies which compared the enzyme activity using Co^2 +^ instead of Mn^2 +^ described a decrease of 89% for murine (Elhammer and Kornfeld, 1986), 39% for porcine (Wang et al., 1992) and 0% for bovine *N*-acetylgalactosaminyltransferases (Takeuchi et al., 1985). Interestingly, the stimulation of xGalNAc-T16 by Ni^2 +^ seems to be much higher compared to xGalNAcT-6 (the latter's activity being only 12% compared to the xGalNAc-T16 conversion rate). Only the xGalNAc-T6 isoform could be stimulated by Zn^2 +^, whereas Ca^2 +^ showed higher stimulation for xGalNAc-T16.

### 3.5 pH dependency of xGalNAc-T6 and -T16

In order to determine the pH optimum of the two recombinant enzymes different buffers were evaluated. Finally, MES-buffer (pH 5.0–7.5) and AMPD-buffer (pH 7.5–9.5) were used to yield reproducible data (in triplicate) for the determination of the pH-optimum (Fig. 4c). Both enzymes show a relative broad pH-optimum between 5.5 and 7.5 with maximal activities at pH 6.0. Below pH 6.0, the enzyme activities dropped steeply, while above pH 7.0, the reduction of the activities dropped over a longer pH range. These results are in agreement with the expected values for enzymes located in the Golgi apparatus, considering that the pH range in this organelle has been estimated between 5.8 and 6.6 (Wu et al., 2001). A comparison with the results on pH-optima described in other studies indicates that both xGalNAcT-isoforms have slightly lower pH-optima than mammalian isoforms, which were determined between 6.5 and 8.2 (Takeuchi et al., 1985; Wang et al., 1992; Mendicino and Sangadala, 1998). Indeed, in our hands, activity tests using the recombinant human isoform 2 and MES-buffer at pH 5, 6 and 7 showed highest conversion at pH 7 (data not shown).

## 4 Discussion

Mucin-type O-linked glycosylation is ubiquitous in animals and is defined as having a ‘core’ GalNAc residue linked to serine or threonine residues, although linkage to tyrosine may also occur rarely (Steentoft et al., 2013). After transfer of GalNAc by *N-*acetylgalactosaminyltransferases, a range of different ‘cores’ can be formed which then can be further elaborated. In amphibians, the O-glycan chains of a range of amphibian egg mucins have been shown in a number of studies to be rather complex and to display a range of unusual and extended oligosaccharide chains (Guerardel et al., 2000). In terms of glycosyltransferases, amongst the few from an amphibian to be previously characterised are two *N-*acetylglucosaminyltransferases (GlcNAc-TI and -TII) and one sialyltransferase (xSTX) involved in N-glycan (and not O-glycan) biosynthesis (Kudo et al., 1998; Mucha et al., 2001, 2002); only recently was a paper including enzymatic data on an amphibian *N-*acetylgalactosaminyltransferase published (Boskovski et al., 2013). On the other hand, enzymes which catalyse the first step in mucin biosynthesis have been studied from a number of mammalian and invertebrate sources; obviously, the first studies were on activities in crude tissue extracts, but interestingly the basic properties (cation dependency and pH optimum) are similar to those defined in the present study (McGuire and Roseman, 1967). There are, of course, a number of other different forms of O-glycosylation based on modification of serine and/or threonine (e.g., O-linked fucose, glucose, xylose and *N-*acetylglucosamine), which also have important roles in development, morphogenesis and signaling (Wilson, 2004; Zachara and Hart, 2006; Takeuchi and Haltiwanger, 2010).

**Fig. 5:**
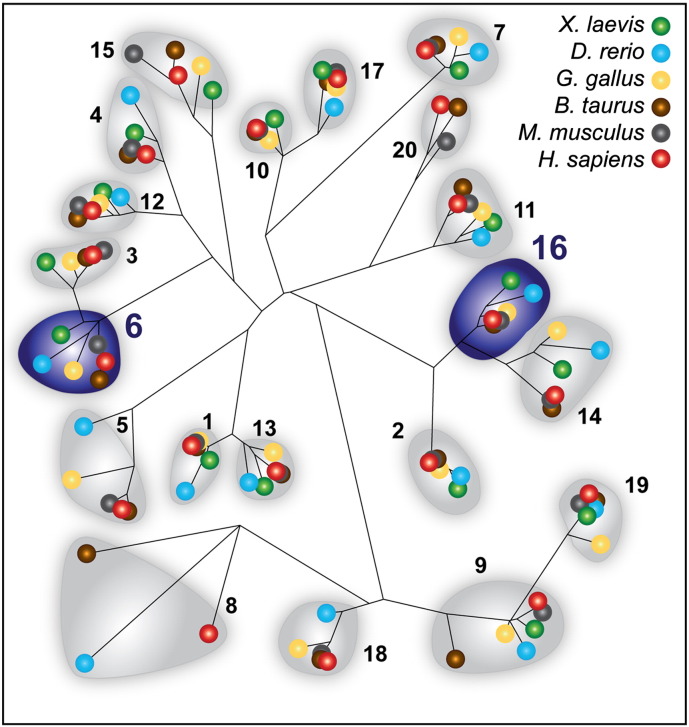
A phylogeny of polypeptide-modifying *N-*acetylgalactosaminyltransferases. The NCBI databank was searched for frog, fish, chicken, bovine, murine and human homologues of GalNAc-T1 through to -T20; frog orthologues of the human T5, T8, T18 and T20 as well as mouse and chicken homologues of T8 were not found in this search. Highlighted are the T6 and T16 groups which are the focus of the current study.

Since the expression of the first recombinant *N*-acetylgalactosaminyltransferases 20 years ago (Hagen et al., 1993), there have been intense efforts to characterize all members of this family (Ten Hagen et al., 2003; Bennett et al., 2012). In the present study, we report the characterization of two recombinant frog GalNAcT isoforms, xGalNAc-T6 and xGalNAc-T16, respectively. Data on the activity and function of human GalNAc-T16 and of one frog enzyme (xGalNAc-T11) were only published during preparation of this manuscript (Raman et al., 2012; Boskovski et al., 2013). In total there are sixteen *N*-acetylgalactosaminyltransferases in frog as shown by an analysis of the whole enzyme family, including those members identified from *Xenopus tropicalis* (Bennett et al., 2012), a number similar to that in *Homo sapiens*, but higher than in invertebrates. Fig. 5 depicts a proposed phylogeny of the enzymes from *X. laevis*, the species from which the xGalNAc-T6 and -T16 in the current study originate, in comparison to the fish, chicken, bovine, murine and human forms. GalNAc-T6 isoforms group with GalNAc-T3, whereas GalNAc-T16 isoforms are close to GalNAc-T2 and -T14; unlike some other isoforms, these can all accept peptides which have not previously been glycosylated, which suggests that they are *N*-acetylgalactosaminyltransferases which act early in mucin biosynthesis.

Due to the high sequence similarity of comparing isoforms of different species, similar biological functions of these enzymes in all vertebrates can be postulated. Compatible with studies on the mammalian isoforms, testing different acceptor substrates revealed that xGalNAc-T6 had broader substrate specificity than xGalNAc-T16; xGalNAc-T6 showed additionally for some substrates the capacity to catalyse the transfer of more than one GalNAc residues to certain peptides. Whereas xGalNAc-T16 transferred only one GalNAc residue to the acceptor peptide (EA2), which was used for the enzyme characterization, xGalNAc-T6 catalysed the transfer to up to three GalNAc residues to EA2 after overnight incubation (Table 1); this at least indirectly indicates that this enzyme also acts on glycopeptides. A possible reason for the differences between the two enzymes might be that EA2 is not related to the primary target peptide for xGalNAc-T16; however, the relative inability of mammalian GalNAc-T16 to multiply glycosylate peptides and its restricted substrate range have also been recently shown (Raman et al., 2012; Kong et al., in press). The mammalian GalNAc-T6 has been previously shown to act on the Muc1 and EA2 peptides (Bennett et al., 1999), whereas the recent data on human GalNAc-T16 has been found to display significant activity towards Muc5, EA2 as well as peptides based on LRP1 (low-density lipoprotein receptor related protein 1) repeats (Raman et al., 2012; Pedersen et al., 2014). The novel substrate used in our study, a peptide based on the sequence of human ActR-IIB, was especially well accepted by xGalNAc-T16 with a singly-glycosylated product being detected; the mammalian enzyme, as coincidentally published during review of this manuscript (Kong et al., in press), also has excellent activity towards a similar sequence, suggesting conservation of both the primary sequence and the enzymatic specificity of GalNAc-T16 during vertebrate evolution. The activity towards the ActR-IIB peptide is of interest considering the previous biological data on xGalNAc-T16.

The two *N*-acetylgalactosaminyltransferases isoforms examined here also have different biological roles; whereas xGalNAc-T16 was shown to inhibit a TGF-β and BMP signaling pathway by O-glycosylation of ActR-IIB (Herr et al., 2008), TGF-β was found to increase expression of the O-glycan-dependent oncofoetal form of fibronectin and induce epithelial-mesenchymal transition in a manner inhibited by siRNA targetting GalNAc-T3 and -T6 (Freire-de-Lima et al., 2011). Our biochemical analysis provides in vitro support the previously proposed role of xGalNAc-T16 in the negative regulation of BMP signaling via ActR-IIB. In particular, our data provides biochemical evidence that GalNAc-T16 might indeed directly and specifically modify ActR-IIB. In addition our data on xGalNAc-T6 indicates that its expression is dependent on active BMP signaling. The microinjection of mRNA encoding xGalNAc-T6 resulted in a slight reduction of anterior structures (Fig. 1), which may imply that xGalNAc-T6 rather promotes BMP activity. However, in loss-of-function experiments using specific morpholino oligonucleotides no significant phenotype could be observed (unpublished data). Thus, the biological function of xGalNAc-T6 remains elusive: nevertheless, xGalNAc-T6 displays a very distinct expression pattern and biochemical activities as compared to GalNAc-T16. Indeed, together with data from others indicating the importance of *X. tropicalis* GalNAc-T11 in Notch-dependent left-right patterning (Boskovski et al., 2013), our study shows that there are distinct roles for different members of this large glycosyltransferase family and that *Xenopus* is a valuable system for examining their contrasting biological functions during early vertebrate embryogenesis. In the future, systematic biochemical and developmental studies on *N-*acetylgalactosaminyltransferases in different systems will certainly further aid our understanding of why there are twenty or so enzymes in vertebrates capable of catalysing what is apparently the same reaction.

## Figures and Tables

**Table 1:** Peptide specificities of the frog xGalNAc-T isoforms. Masses indicated with (*m/z*) correspond to the sodium adduct of the peptides with those of products of successful glycosylation reactions in bold; the indices beside the rounded *m/z* values indicate the number of transferred GalNAc residues per peptide after 16 h of incubation. The asterisk indicates that these species were detected only in low amounts. RNApII and neuroglycan peptides are acceptors for O-GlcNAc and O-xylosyl transferases, but were demonstrably not substrates for either enzyme.

Peptide	*m/z*	Peptide sequence	xGalNAc-T6 (*m/z*)	xGalNAc-T16 (*m/z*)
Muc1fr1	1023	GAPGSTAPPAGK	**1235**^1^	1023
Muc1fr2	1075	GAHGVTSAPAGK	**1278**^1^	1075
Muc1fr3	1134	GAAPDTRPAAGK	1134	1134
OPSS1	1285	GAGAPGPTPGPAGAGK	**1488**^1^	**1488**^1^
OPSS4	789	*Ac*PTPGPAGK	**992**^1^	**992**^1^
EA2	1340	PTTDSTTPAPTTK	**1746**^2^, **1949**^3^	**1543**^1^
ActR-IIB	1566	GPEVTYEPPPTAPTL	**1769**^1⁎^, **1972**^2⁎^	**1769**^1^
RNApII	1045	YSPTSPSKR	1045	1045
Muc5Ac	1555	GTTPSPVPTTSTTSAK	**2165**^3^, **2368**^4^	**1758**^1^
Neuroglycan	1340	VTAEAGSGDAQTALK	1340	1340
